# Voice and identity: reflections on vocal congruence in transgender and laryngectomized people

**DOI:** 10.1590/2317-1782/e20250173en

**Published:** 2026-01-26

**Authors:** Nair Kátia Nemr, Rodrigo Dornelas

**Affiliations:** 1 Curso de Fonoaudiologia, Faculdade de Medicina, Universidade de São Paulo – USP - São Paulo (SP), Brasil.; 2 Departamento de Fonoaudiologia, Faculdade de Medicina, Universidade Federal do Rio de Janeiro – UFRJ - Rio de Janeiro (RJ), Brasil.

Dear editors,

We would like to begin this letter with a thought that may seem unlikely at first: What do transgender and laryngectomized people expect from their voices? The complexity of this question invites us to reflect on realities that, although distinct, share profound challenges related to voice, identity, and communication.

Would these two experiences possibly share points of convergence or tension?

Violence against transgender people in Brazil and worldwide transcends the bounds of civility. A world that overvalues ​​binary thinking to the detriment of diversity perceives any attention paid to anything that questions or deviates from the norm as uncomfortable and embarrassing. Likewise, the curiosity with and a certain aversion to tracheostomies resulting from total laryngectomy, as well as the impression that these people are deaf because they cannot communicate vocally, are also uncomfortable and embarrassing. Thus, both cases converge in compromised oral, verbal, and nonverbal communication.

Total laryngectomy poses the challenge of coming to terms with a completely new voice (when possible) because of a surgery necessary for survival. In turn, transgender people often feel disconnected from the unaltered voices their bodies produce^(1)^. This difference between voice and perceived identity raises important questions about vocal congruence. It highlights how the unique and social experiences of different groups influence their self-image and the search for voices that reflect their desired identity. Notably, quality of life related to voice and communication is more impaired in transgender people than in those who have undergone laryngectomies^(1)^. Is this surprising?

This observation led us to reflect: If a transgender person is identified as such by their voice or by the movement of the laryngeal prominence during swallowing (a physiological action), their life may be at risk in extreme cases. People who have undergone laryngectomies have chosen to permanently lose their laryngeal voice to regain their lives, as a kind of rebirth after a serious illness.

Hence, in both cases, communication and life can be considered as a single path, moving in the same direction, but in an inversely proportional relationship. For transgender people, the presence of the former can compromise the latter; for laryngectomized people, the absence of the former is the price to pay for preserving the latter.

On second thought, any alternative means of communication for laryngectomized individuals – whether by exceptionally learning a new way of communicating or by using a tracheoesophageal prosthesis, esophageal speech, or an electronic larynx – can, at best, approximate laryngeal voice, but never completely replace it. When they occlude the tracheostoma, activate the electronic larynx, or perform the maneuver to introduce air into the esophageal tract, listeners also pay attention to the gesture. The impact of treatment can pose even greater challenges for women with laryngectomies.

However, this may not be enough to minimize the impacts on transgender people’s quality of life, even if their vocal frequency is compatible with the gender with which they identify (as perceived by listeners), and their expressiveness is in harmony.

According to Crow et al.^(2)^, changes in identity perception are often related to significant changes in voice, as observed in groups such as laryngectomized patients, although many report being satisfied with the alternative communication methods they have achieved and feel fortunate to have survived cancer. Transgender people may differ from this group, as they intentionally seek modifications to achieve a comfortable identity that represents them. Voice is one of the main obstacles to identity and poses a challenge during transition. Incongruence is present in both examples through different pathways.

Congruence between vocal quality and self-designation needs to be established and maintained to cope with changes in identity perception. This congruence is essential for individuals to feel secure in their identity, whether facing challenges related to gender affirmation (among transgender people) or searching for a new means of communication that reestablishes social interaction (among laryngectomized individuals)^(3-5)^.

It's important to discuss some aspects of communicative intent and habits that influence vocal production and communication, which may overlap between these two populations.

What do laryngectomized and transgender individuals have in common when it comes to vocal construction?

Smoking often contributes to the development of cancer in people who have undergone laryngectomies. Interestingly, some transgender people intentionally start this habit to modify their voices – transgender men do so to reach lower frequencies, and transgender women smoke to introduce vocal fry or other noises that soften the pitch of the voice, depending on the person's body structure.

Some transgender women undergo risky procedures (e.g., injecting industrial silicone into the neck without medical supervision) to disguise the laryngeal prominence, which is removed in a total laryngectomy.

Vocal intensity is also similar between the two groups; it is weaker in laryngectomized people, while transgender women control it to avoid attracting attention. Transgender men may lose some decibels due to hormone treatment, which causes structural changes in the vocal folds.

Both groups prefer not to talk on the phone, especially after the popularization of voice messaging via communication apps. People who have had laryngectomies avoid this technology for fear of not being intelligible; transgender people, on the other hand, avoid sending audio messages lest their voices might reveal that they are trans.

The therapeutic experience of speech-language-hearing pathologists is genuinely enriched by understanding how people see and understand themselves, and by recognizing and embracing these different ways of being in the world. Life becomes much deeper when we have the opportunity to interact with people of all backgrounds and experiences. This openness to embracing diversity, which challenges norms, including patriarchal ones, is a valuable contribution to the world.

Thus, we believe that this reflection, which brings two historically silenced groups into dialogue, can broaden perspectives and spark fruitful discussions regarding voice and communication by illuminating points of contact, differences, and tensions between the experiences of transgender and laryngectomized people. We hope to contribute to a more sensitive, ethical, and politically committed clinical practice that listens to all voices, including those that challenge established standards.
