# Supplementary material for: Voice and identity: reflections on vocal congruence in transgender and laryngectomized people
Source: Codas. 2026 Jan 26;38(1):e20250173. doi: 10.1590/2317-1782/e20250173en (PMC12978315; doi:10.1590/2317-1782/e20250173en)
Supplement: Translation

# Translation: Voz e identidade: reflexões sobre congruência vocal em pessoas transgênero e laringectomizadas

**DOI:** 10.1590/2317-1782/e20250173pt

**Authors:** Nair Kátia Nemr, Rodrigo Dornelas

**Affiliations:** 1 Curso de Fonoaudiologia, Faculdade de Medicina, Universidade de São Paulo – USP - São Paulo (SP), Brasil.; 2 Departamento de Fonoaudiologia, Faculdade de Medicina, Universidade Federal do Rio de Janeiro – UFRJ - Rio de Janeiro (RJ), Brasil.

Prezadas editoras,

Gostaríamos de iniciar esta carta com uma provocação que pode parecer, à primeira vista, improvável: o que pessoas transgênero e pessoas laringectomizadas esperam de suas vozes? A complexidade dessa pergunta nos convida a refletir sobre realidades que, embora distintas, compartilham desafios profundos relacionados à voz, identidade e comunicação.

Seria possível encontrar pontos de convergência ou até mesmo tensões entre essas duas experiências?

A violência contra pessoas trans, tanto no Brasil quanto no mundo, ultrapassa os limites da civilidade. Um mundo que supervaloriza o pensamento binário em detrimento à diversidade enxerga como incômoda e constrangedora qualquer atenção dada a algo que questione ou fuja da normatividade. E a curiosidade e certa aversão à traqueostomia resultante da laringectomia total ou a impressão de que essas pessoas são surdas por não se comunicarem por meio da voz também são incômodas e constrangedoras. Assim, ambos os casos convergem em uma comunicação oral, verbal e não verbal comprometida.

A laringectomia total impõe o desafio de se reconciliar com uma voz completamente nova (quando isso é possível), consequência de uma cirurgia necessária para a sobrevivência. Por sua vez, pessoas trans frequentemente se sentem desconectadas das vozes inalteradas que seus corpos produzem^(1)^. Essa diferença entre voz e identidade percebida levanta questões importantes sobre a congruência vocal. Ela evidencia como as experiências singulares e sociais de diferentes grupos influenciam sua autoimagem e a busca por vozes que reflitam a identidade desejada. É notável que a qualidade de vida relacionada à voz e à comunicação seja mais prejudicada em pessoas trans do que em pessoas laringectomizadas^(1)^. Isso surpreende?

Essa constatação nos levou a uma reflexão: se uma pessoa trans é identificada como tal por sua voz ou pelo movimento da proeminência laríngea durante a deglutição (uma ação fisiológica), sua vida pode estar em risco, em casos extremos. Já pessoas laringectomizadas optaram por perder permanentemente a voz laríngea para recuperar a vida – como uma espécie de renascimento após uma doença grave.

Essa reflexão nos leva a considerar a comunicação e a vida, em ambos os casos, como um mesmo caminho, seguindo na mesma direção, mas em uma relação inversamente proporcional. Para pessoas trans, a presença da primeira pode comprometer a segunda; para pessoas laringectomizadas, a ausência da primeira é o preço para preservar a segunda.

Em uma segunda reflexão, qualquer alternativa para a comunicação de pessoas laringectomizadas, seja por meio de um processo de aprendizagem excepcional de um novo modo de se comunicar ou com uma prótese traqueoesofágica, fala esofágica e/ou uso de laringe eletrônica, pode, no melhor dos casos, se aproximar da voz laríngea, mas jamais substituí-la por completo. Quando elas ocluem o traqueostoma, ativam a laringe eletrônica ou realizam a manobra para introduzir ar no trato esofágico, os ouvintes também prestam atenção ao gesto. O impacto do tratamento pode representar desafios ainda maiores para mulheres laringectomizadas.

Quanto às pessoas trans, mesmo que a frequência vocal esteja compatível com o gênero com o qual a pessoa se identifica, como percebido por outros ouvintes, e que a expressividade esteja em harmonia, isso pode não ser suficiente para minimizar os impactos em sua qualidade de vida.

Segundo Crow et al.^(2)^ mudanças na percepção da identidade estão frequentemente relacionadas a mudanças significativas na voz, como observado em grupos como os pacientes laringectomizados, embora muitos relatem estar satisfeitos com os métodos alternativos de comunicação que conquistaram e se sintam felizes por terem sobrevivido ao câncer. Pessoas trans podem se diferenciar desse grupo, uma vez que buscam intencionalmente modificações para alcançar uma identidade confortável que as represente. A voz é um dos principais obstáculos à identidade e representa um desafio na transição. A incongruência está presente em ambos os exemplos, por caminhos distintos.

A congruência entre a qualidade vocal e a autodesignação precisa ser estabelecida e mantida para lidar com as mudanças na percepção da identidade. Essa congruência é fundamental para que a pessoa se sinta segura em relação à sua identidade, seja no enfrentamento dos desafios relacionados à afirmação de gênero no caso de pessoas trans, seja na busca por um novo meio de comunicação que restabeleça a interação social para pessoas laringectomizadas^(3-5)^.

É pertinente discutir alguns aspectos da intenção comunicativa e dos hábitos que influenciam a produção vocal e a comunicação, os quais podem se sobrepor entre essas duas populações.

O que pessoas laringectomizadas e pessoas trans têm em comum na construção vocal?

O tabagismo frequentemente contribui para o desenvolvimento do câncer em pessoas laringectomizadas; curiosamente, algumas pessoas trans adotam esse hábito intencionalmente para modificar suas vozes. Em homens trans, busca-se deliberadamente alcançar frequências mais graves; no caso de mulheres trans, o cigarro pode introduzir o *vocal fry* ou outros ruídos que suavizam o *pitch* da voz, de acordo com a estrutura corporal da pessoa.

Para disfarçar a proeminência laríngea, que é removida na laringectomia total, algumas mulheres trans se submetem a procedimentos arriscados, como a injeção de silicone industrial no pescoço, sem supervisão médica.

A intensidade vocal também é semelhante entre os dois grupos, mais fraca em pessoas laringectomizadas, enquanto mulheres trans controlam sua intensidade vocal para não chamar atenção. Homens trans podem perder alguns decibéis devido ao tratamento hormonal, que provoca mudanças estruturais nas pregas vocais.

Ambos os grupos preferem não falar ao telefone, especialmente após a popularização do envio de mensagens de voz por aplicativos de comunicação. Pessoas laringectomizadas evitam essa tecnologia por receio quanto à inteligibilidade; já pessoas trans evitam enviar áudios que possam revelar, pela voz, que se trata de uma pessoa trans.

A experiência terapêutica de fonoaudiólogas e fonoaudiólogos é genuinamente enriquecida ao compreender como as pessoas se veem e se entendem, e ao perceber e acolher essas diferentes formas de estar no mundo. A vida ganha muito mais profundidade quando temos a chance de interagir com pessoas de todas as origens e vivências. Essa abertura ao acolhimento da diversidade, que desafia normas, inclusive as patriarcais, é uma valiosa contribuição para o mundo.

Por isso, acreditamos que esta reflexão, que coloca em diálogo dois grupos historicamente silenciados, pode ampliar olhares e provocar discussões frutíferas no campo da voz e da comunicação ao iluminar pontos de contato, diferenças e tensões entre as vivências de pessoas trans e laringectomizadas. Assim, esperamos contribuir para uma prática clínica mais sensível, ética e politicamente comprometida com a escuta de todas as vozes – inclusive daquelas que desafiam os padrões estabelecidos.
